# The making of local hospital discharge arrangements: specifying the role of professional groups

**DOI:** 10.1186/s12913-015-0963-4

**Published:** 2015-08-04

**Authors:** Viola Burau, Flemming Bro

**Affiliations:** CFK – Public Health and Quality Improvement, Olof Palmes Allé 15, 8200 Aarhus N, Denmark; Department of Political Science, Aarhus University, Aarhus N, Denmark; Department of Public Health, Research Unit for General Practice, Aarhus University, Bartholins Allé 2, 8000 Aarhus C, Denmark

**Keywords:** Hospital discharge, Professional groups, Organisational change, Local contexts

## Abstract

**Background:**

Timely discharge is a key component of contemporary hospital governance and raises questions about how to move to more explicit discharge arrangements. Although associated organisational changes closely intersect with professional interests, there are relatively few studies in the literature on hospital discharge that explicitly examine the role of professional groups. Recent contributions to the literature on organisational studies of the professions help to specify how professional groups in hospitals contribute to the introduction and routinisation of discharge arrangements. This study builds on a view of organisational and professional projects as closely intertwined, where professionals take up organising roles and where organisations shape professionalism.

**Methods:**

The analysis is based on a case study of the introduction and routinisation of explicit discharge arrangements for patients with prostate cancer in two hospitals in Denmark. This represents a typical case that involves changes in professional practice without being first and foremost a professional project. The multiple case design also makes the findings more robust. The analysis draws from 12 focus groups with doctors, nurses and secretaries conducted at two different stages in the process of the making of the local discharge arrangements.

**Results:**

From the analysis, two distinct local models of discharge arrangements that connect more or less directly to existing professional practice emerge: an ‘add-on’ model, which relies on extra resources, special activities and enforced change; and an ‘embedded model’, which builds on existing ways of working, current resources, and perspectives of professional groups. The two models reveal differences in the roles of professional groups in terms of their stakes and involvement in the process of organisational change: whereas in the ‘add on’ model the professional groups remain at a distance, in the ‘embedded model’ they are closely engaged.

**Conclusions:**

In terms of understanding the making of hospital discharge arrangements, the study contributes two sets of insights into the specific roles of professional groups. First, professional interests are an important driver for health professionals to engage in adapting discharge arrangements; and second, professional practice offers a powerful lever for turning new discharge arrangements into organisational routines.

## Background

Over the last several decades, there has been an increasing focus on discharging hospital patients more quickly. This reflects economic pressures arising from a combination of advances in medical technology, demographic changes and, increasingly, scarce resources for welfare spending [1, 2], together with New Public Management reforms and their concern for greater efficiency [3]. At the same time, technological, organisational, medical and pharmaceutical progress also means that hospitals are no longer the only location for delivering medical care [2]. Indeed, timely discharge has emerged as one of the key components of contemporary hospital governance [2].

The focus on timely discharge includes not only patients who have had surgery, but also patients who are in programmes following a major illness like cancer. There is growing evidence that follow-up programmes based in hospitals do not necessarily work well: studies show that they can have a poor record of detecting the recurrence of illness and do not improve survival rates [4–6]. Other studies have found that the follow-ups do not meet patient needs, for example related to dealing with the side effects of treatment and the fear of reoccurrence of the illness [7, 8]. Moreover, patients can also experience the continued contact to hospitals as stressful. Taken together, this has prompted reviews of follow-up programmes with one strategy to move programmes out of hospitals as part of shared care arrangements [9–12].

The organisation of explicit discharge arrangements is one milestone in shared care arrangements for follow-up programmes which typically involves changes in professional practice across different medical specialties and/or health professions, including administrative staff within hospitals. The issue is who takes on the new tasks of identifying patients, coordinating patient discharge and auditing the discharge letters. Altering discharge arrangements, therefore, involves organisational changes which closely intersect with professional interests. The literature on hospital discharge acknowledges the importance of professional groups for the organisational changes involved [13–17] by examining the views of health professionals in relation to a range of issues: the challenges and solutions of organisational processes [13], outcomes [14], the quality of discharge arrangements [15, 17] and guidelines [16]. However, the literature says little about the specific roles played by professional groups. Recent contributions to the literature on organisational studies of the professions help specify how professional groups in hospitals contribute to the introduction and routinisation of discharge arrangements.

The underlying idea is that professionalism is part of a complex set of governance arrangements, that include hierarchical and (possibly market) forms of governing as well as professional self-regulation [18–21]. Going beyond the traditional dualism between professions and organisations/management, professionalism and managerialism are conceptualised as two different modes of governing, one ‘internal’ and the other ‘external’, that are concerned with improving the control over professional knowledge. Following this, Munzio and colleagues [22, 23] argue that professional projects closely interact with institutional projects of the organisation and thus are embedded in negotiated settlements. Indeed, professions are important actors in organisational change, in as much as organisations need to accommodate professional practice. Building on this view of interdependence, Noordegraaf [24] introduces the notion of ‘organized professionalism’, where professionals take up organising roles and where organisations structure professionalism.

From this perspective, in the move toward explicit discharge arrangements, professional and organisational projects intersect and this mobilises professional groups, who, in conjunction with management, adapt the generic model of discharge arrangements to the local context of the individual outpatient department. This occurs through interpreting and translating the specific changes in the work flows into local models [25]. The present analysis is built on a case study of the introduction and routinisation of explicit discharge arrangements for patients with prostate cancer in two hospitals in Denmark and addresses the following questions: What are the specific roles of professional groups in the making of local models of discharge arrangements and how does the involvement of professional groups affect the introduction and routinisation of discharge arrangements?

## Methods

### From generic to local discharge models

The study was concerned with the process of the making of the local model of explicit discharge arrangements. The starting point was a generic model for discharge of patients from hospital to general practice developed by the steering group of the project. Based on national guidelines, the group produced a one-page summary sheet detailing which patients to discharge for follow-up by general practitioners (GPs). This information provided the basis for the three components of the generic model: first, a guideline for identifying patients suitable for discharge; second, a personalised discharge leaflet with information about procedures for control/treatment in general practice together with the criteria for readmission; and, finally, a standard form for discharge letters, to ensure GPs receive all relevant information. Following the theory on complex interventions, the project contained specified, core elements, whereas a ‘soft periphery’ was left to local adaptation [26].

The literature on organisational change often treats such a process as a more or less linear sequence of different phases [27]. In contrast, May and Finch [28] describe organisational change more broadly as the social organisation of bringing new action into practice. The view of a more iterative process fits very well with an organisational studies perspective on professions, which is concerned with micro level processes that occur as part of day-to-day practice in conjunction with management. Within this framework, the analysis looked at processes of introducing and routinising explicit discharge arrangements.

### Case study design

The analysis was based on a case study [29] of a six-month project that introduced explicit discharge arrangements for patients with stable forms of prostate cancer to general practice for follow-up controls and further treatment. This represented a typical case of organising explicit discharge arrangements, as it involved changes in professional practice without being primarily a professional project. The findings of this study, therefore, can say something about the average case of organising discharge in health services. The analysis involved a multiple case design that included two hospitals in the region of Central Denmark, thus, making the findings more robust. The two cases were selected following the logic of literal replication [29] where the expectation is that the cases produce similar findings. In this study, the expectation was that professional groups would be involved in the making of local models of discharge arrangements in broadly similar ways since the two departments are part of the same regional healthcare system and related funding arrangements and they have a comparable patient population as well as the same mix of doctors, nurses and secretaries with similar educational backgrounds.

### Data collection

The analysis was based on material gathered as part of two sets of focus groups, each with the main professional groups in the outpatient department in the two hospitals: doctors, nurses and secretaries, making for 12 groups in total. Focus groups rely on in-group interaction and discussion, therefore they represent a highly suitable method of data gathering for an analysis of the *collective* views/actions of professional groups [30, 31]. In order to capture the iterative processes of introducing and routinising explicit discharge arrangements, the focus groups were conducted about 3 months into the project and about 2 months after the end of the project. The first author conducted all focus groups with the help of two research assistants. Focus groups can vary in size, and some studies have found that small focus groups run more smoothly [30]. In the present study, the focus groups included an average of 3 participants, since only a small share of the staff of the outpatient department was involved in the project. The total number of participants was 37 and in each of the second set of focus groups there was at least one participant who also participated in the first focus group. The focus groups lasted between 30 and 40 min and were based on a set of broad questions relating to operationalisation of the different parts of the analytical framework (see Table 1 below for an overview).

**Table 1:** Operationalisation of analytical framework

LOCAL MODELS OF DISCHARGE ARRANGEMENTS	Identifying patients
	Discharging patients
	Writing discharge letters
ACTORS	
Interests of professional groups	Professional interests in discharge arrangements
Resources of professional groups	
	Dealing with uncertainties about new work flow
PROCESSES	
Introducing and routinising discharge arrangements	Formal processes: support by management, use of project material
	Informal processes of interpretation/translation

The analytical framework consisted of three components: the local models of discharge arrangements, actors and processes. The indicators for the local models reflect the cornerstones of the professional/organisational practice, notably identifying patients, discharging patients and writing discharge letters. Concerning actors, and following the organisational studies perspective on professions, the interests of professional groups originate from their respective jurisdictions, but are negotiated in the context of the individual department. A relevant indicator is the extent to which the professional groups see the organisational change as congruent with their interests and in what specific substantive respects this occurs. Interests are likely to be defined broadly and concern the organisation, the patient and professional practice. The resources available to individual professional groups consist of the ability to exercise power and reflect the respective position in the division of labour in the outpatient department as well as the opportunities offered by management in the process of organisational change. Because uncertainties potentially mobilise interests and power resources, a relevant indicator is how individual professional groups deal with uncertainties about the new work flow. The process of making of discharge arrangements is concerned with the initial introduction and later routinisation of specific work flows. Following the organisational studies perspective on professions, this includes both formal processes such as support by management and use of process documents, and informal processes where professional groups interpret and translate discharge arrangements as part of their day-to-day practice.

### Data analysis

The focus groups were recorded, transcribed verbatim and approved by the participants. Danish legislation requires no ethical approval for this type of study (see Act on Research Ethics Review of Health Research Projects, Law no. 593, 14 June 2011; http://www.cvk.sum.dk/English/actonabiomedicalresearch.aspx). The specific guidelines on qualitative studies state that ‘questionnaire-based examinations shall be treated like the so-called register research projects, i.e. that they have to be notified ONLY if the project will include examination of human biological material or examination of individuals (…) Interview examinations are comparable to questionnaire-based examinations’ (Section 2.8, Guidelines about Notification etc. of a Biomedical Research Project to the Committee System on Biomedical Research Ethics, No 9154, 5 May 2011; http://www.cvk.sum.dk/English/guidelinesaboutnotification.aspx). All respondents gave their formal oral consent for recording focus group proceedings and approved the text of the written and anonymised transcript of the focus group that was used for the analysis.

The analysis followed a thematic approach which aims to identify common threads that extend across a set of interviews [32]. The approach typically combines deductive and inductive elements. The analysis began by constructing and applying a set of initial codes derived from operationalisation of the analytical framework. The emerging codes were then collated into potential themes, which subsequently were reviewed and refined. This included a triangulation with the qualitative process evaluation based on the meetings with the project group. The first author took the lead on the analysis, whereas the second author contributed with critical feedback. This occurred on an ongoing basis and the iterative nature of the analysis allowed the two authors to work towards a truly joint analysis.

## Results

### Analysis of hospital A

#### The local model: an add-on model which gets slimmed down

In Hospital A, the outpatient department was medium sized and the majority of the consultations were conducted by doctors. As part of the initial introduction of discharge arrangements, the department adopted a local model that was added onto existing professional practice under which a project nurse was instrumental in facilitating key processes of working within the new arrangements. The project nurse went through the patient list of the department each week in advance and identified potential patients for discharge, who were then approved by the leading consultant. The project nurse attached the leaflet with patient information to the patient file which served as a double reminder for the individual doctors conducting the consultations with patients, namely to decide if the patient indeed could be discharged, and to pass on the leaflet with patient information. However, in relation to the specific clinical decision to discharge the patient, the streamlining of work flows co-existed with the professional judgment of individual doctors.

Another element of the new work flow was that a nurse should sit in on the consultation to listen to what was being said and to assess if the patient understood the information provided. If necessary, the nurse followed up with the patient to address any queries, repeated any information where needed and initiated any follow-up activities. Immediately after the end of the consultation, the doctor dictated the discharge letter, which was then typed by a medical secretary. In Hospital A, the standard form for the discharge letter was integrated into the system for electronic patient records, making it obvious when information was missing. At the beginning of the project period, the secretaries passed on all draft discharge letters to the project nurse, who then discussed any queries with the doctor concerned. Indeed, this was contrary to normal practice when dealing with queries, as one of the secretaries explained:

‘In relation to all other types of written communication we go directly to the doctor [concerned]. However, [in relation to the discharge project] she [the project nurse] acts as an intermediary […]. It appears she wants to be involved [in clarifying queries] so that she in some ways determines, how [the discharge project] develops.’ (Secretary, Hospital A)

The fact that the local model for discharge in Hospital A was added to existing professional practice seemed to make it more difficult to establish routines, thus, after the completion of the project period the local model was slimmed down in significant ways. The centralised identification of patients was replaced by the ‘old’ procedure where individual doctors used their individual professional judgement only. As part of the discharge, the doctors no longer handed out the leaflet with patient information, and the oral communication with the patient and the discharge letter sent to the general practitioner was considered sufficient. However, the doctors continued to provide information that was needed by the secretaries to write a standard discharge letter.

#### Professional groups: mixed interests and little mobilisation of resources

The professional groups in Hospital A had highly varied interests in the new discharge arrangements. Interests varied both in terms of how directly they were related to professional interests and whether the overall (professional) interest was positive or not. The doctors acknowledged the specific advantages discharge arrangements might have for the department at large (reducing the number of control appointments and, thereby, freeing resources for other patients) and for patients (receiving the message that their cancer is stable and saving visits to hospitals). However, during as well as after the project period, the doctors largely distanced themselves from the streamlined process. Moreover they kept control of the decision about who to discharge through their individual professional judgment:

‘What is a stable PSA [prostate specific antigen]? […] This is a matter of professional judgement. There is no standard answer, where one can say, “if the result is this, the patient falls into one treatment category, and if not, the patient falls into another treatment category”.’ (Doctor, Hospital A).

After completion of the project they also rejected the routine use of the patient information leaflet. Referring to the project as ‘bureaucratic’, the doctors further distanced themselves from the organisational changes as initially envisaged, contrasting it with their own ‘common sense’ approach.

The views of the nurses were also negative. The nurses saw discharge arrangements first and foremost as an unwelcome expansion of their professional territory:

Nurse 1 ‘Professional gains [from the discharge arrangements]? I do not see any. This [that the nurses take part in the patient consultations] is only practical support. Honestly.’

Nurse 2 ‘Many times, […] I feel that the doctor could as well have conducted the patient consultation on his own.’ (Nurses, Hospital A)

More specifically, the nurses did not feel that sitting in on the patient consultations was professionally meaningful but rather an additional responsibility imposed externally by the project.

In contrast to the doctors and the nurses, the secretaries felt that the discharge arrangements were directly in line with their professional interests:

Secretary 1 ‘I feel this [the standard form for discharge letters] is very good, because it helps reducing mistakes.’ […]

Secretary 2 ‘This way one can be sure, that all [the information] is there.’ (Secretaries Hospital A)

By connecting the use of the standard form to the possibility of avoiding mistakes, the secretaries put the discharge arrangements at the centre of their professional practice.

How uncertainties about the new work flow were dealt with offers insights into the power resources of the individual professional groups. The doctors used competitive power associated with clinical judgment to deal with uncertainties emerging as part of the explicit discharge arrangements, namely if a particular patient should be discharged. This involved addressing uncertainties strictly at an individual level. In contrast, although there were corresponding uncertainties for nurses and secretaries, they did not act on them. For nurses, the ambiguities centred on whether they should take part in the patient consultations with doctors, while for the secretaries it was how to deal with draft discharge letters that did not conform to the standard form. Interestingly, although both professional groups had clear negative/positive professional interests in discharge arrangements, neither group mobilized any power resources to pursue their interests: the nurses did not take up their concerns with the leading nurse or the project nurse, and, the secretaries tried to compensate by making the text dictated by doctors fit into the standard form.

#### Process of organisational change: detached from professional groups

The highly mixed interests and the very limited mobilisation of resources distanced professional groups from the process of organisational change. Moreover, this was exacerbated by limited formal processes. The introduction to the project by management was highly varied among the professional groups: doctors received information only after project started, whereas the nurses were uncertain about the type of introduction they were offered. In contrast, the secretaries were only informed about the project by email. The secretaries, especially, highlighted the subsequent knock-on-effects:

‘There was some ‘trial-and-error’ [approach] in the beginning [of the project], and maybe this could have been avoided by including the whole department [in a formal introduction]. (Secretary, Hospital A)

Considering that the secretaries sat at the bottom of the ‘collaborative chain’ it was not surprising that they were particularly concerned about the continuity of work processes.

Also, after the initial introduction the support by management was provided primarily by the project nurse who had to deal with specific queries as they arose, but there were no planned follow-up activities during the project period. The staff was not involved in any processes of adapting the patient leaflet or adjusting the work flow and, thus, they had no sense of ownership:

‘I feel [the fact] that […] it is printed [on the leaflet with patient information], that if the PSA increases with 20 per cent [general practitioners should refer patients back to the hospital] […] is stupid. Here I allow myself to write a number [that is the absolute number of the increased PSA].’ (Doctor, Hospital A)

Significantly, the doctors did not take any active steps to get the form changed or produce a local version of the form.

Interestingly, only the secretaries emphasised the importance of informal processes of interpreting discharge arrangements as part of day-to-day practice.

‘Yes [among us secretaries we talk about the project]. After all, we simply must find out how to make this [the discharge arrangements] work […].’ (Secretary, Hospital A)

As the secretaries explained, this also reflected the fact that they felt that the discharge arrangements came in through the back door. The doctors referred to similar informal processes, but those were more specifically related to individual patients and typically resolved bilaterally between two doctors.

The impression of a process of organisational change that was detached from the professional groups concerned was strongly underscored when viewing the process of routinising discharge arrangements with the project nurse out of the picture. Neither management nor any of the professional groups expressed any explicit responsibility for securing the continuation of discharge arrangements. Although doctors and nurses talked about how to continue with identifying/selecting and discharging patients, the discussions have remained limited. As one doctor explained:

‘[W]e [among doctors] have also subsequently talked about this [discharging patients], that we should continue with discharging them [patients]. The question is if we indeed do this. (Doctor, Hospital A)

This suggests that the continuation of discharge arrangements was first and foremost discussed in normative terms as something doctors should be doing, whereas there was less talk about how this best could be done in practice. Similarly, among the nurses adapting the folder with patient information was mainly discussed as a possibility and the question of who should be in charge remained open.

‘I feel if there was time, one could get together and write such a folder [with patient information] […] that was specific to our department. […] [B]ut there is no progress on this. This is not something one has given priority.’ (Nurse, Hospital A)

This quote clearly indicates that the nurses did not feel any ownership of discharge arrangements. Taken together, the absence of both formal support by management and informal processes among the professional groups resulted in a considerable slimming down of the local model of discharge, which, significantly, occurred by default rather than design.

### Analysis of hospital B

#### The local model: an embedded model that continues

In the local model in Hospital B, the individual processes associated with the discharge arrangements were embedded in existing inter-professional practices in the outpatient department. The department was large and nurses ran their own clinics for selected groups of patients, although they could not discharge patients. Most patients were seen by both a doctor and a nurse and, when present, the role of nurses included not only following up any queries, but also reminding the doctor of the department’s standard procedures including those related to the new discharge arrangements. Although the discharge process remained the responsibility of the doctors, the nurses took on the role as coordinator of procedures in line with their role in relation to other patient groups. One nurse suggested that this reflected mutual expectations both professional groups have about the role of nurses:

‘We [nurses] are all too eager to take on responsibility, but the others [doctors] are also used to this. So, if we did not do this […], this [the discharge project] would not have started so quickly and would not have advanced so much.’ [Nurse, Hospital B]

Here, in contrast to Hospital A, the nurses undertook a larger role of ‘process coordinators’; this role was not new to them and became an integrated part of daily professional practice for all nurses in the department within the first month of the project.

Since only doctors could discharge patients, initially it was decided that doctors should be responsible for identifying potential patients. Subsequently, this went through a series of changes. In practice (and subsequently also formally), initiating this process became primarily the responsibility of nurses. Nevertheless, the process of identifying patients itself developed as a joint activity of nurses and doctors. The involvement of nurses was straightforward for those patients seen by doctors, but patients seen only by the nurses could not be discharged immediately. Furthermore, doctors did not review the list of patients for nurse-led consultations and only doctors could discharge patients. Instead, the nurses booked patients who they considered to be candidates for discharge for a doctor-led consultation as part of the next appointment. However, this meant that all doctors and nurses in the outpatient department were involved in identifying patients across many locales since it was integrated into existing professional practice. The formal criteria for identifying patients, therefore, became ‘owned’ by the nurses and doctors in the outpatient department.

At the beginning of the project, the writing of the discharge letter was not supported by an electronic version of the standard form (either as a macro or as part of the system of electronic patient records) and information was often missing. In contrast to Hospital A, there also appeared to be little informal auditing by the secretaries, as the following exchange illustrated:

Interviewer: ‘What do you do, when the doctors [in the text they dictate] do not follow the standard form [for the discharge letter]?

Secretary 1: ‘I would say nothing. […] In the text [dictated by the doctor] they [the patients] are discharged to general practice and this is the end of the matter. We do not ask them [the doctors] to look again [at the text dictated] and to make changes. At least I have not done this.’

[Secretary, Hospital B]

In the first month of the project period, the monitoring results provided by the project coordinators showed that the quality of the discharge letters was poor and, in response, written instructions templates were placed in the consultation rooms and the standard form was integrated into the system of electronic patient records.

The embedded nature of the local model in Hospital B seemed to offer highly favourable conditions for establishing the local model as a routine. The local model built on existing inter-professional practice, with individual doctors and nurses responsible for identifying patients and with individual nurses acting partly as ‘process coordinators’ in relation to the discharge of patients. The local model continued to exist without any significant changes after completion of the project.

#### Professional groups: direct and positive professional interests and mobilisation of resources

In contrast to Hospital A, all professional groups in Hospital B seemed to feel that discharge arrangements were in line with their respective professional interest. One of the doctors explained that the outpatient department had a long-standing discussion when it was necessary to retain the control of patients with prostate cancer, and continued:

‘I feel that we [doctors in the department] have talked about, that we in one way or another should move the control [of patients] outside [the hospital]. So this is a good project to help doing this.’ (Doctor, Hospital B)

Discharge arrangements emerged as a welcome catalyst for organisational change, which the doctors themselves had talked about for some time. In contrast to Hospital A, the doctors did not use their clinical power to obstruct the change process, but accepted the new discharge arrangement:

‘Well, I feel this [discharge arrangements] is part of us [the doctors?] now. I do not feel there is any problem; we just do it [work according to discharge arrangements]. This is also because we have to; we are very hard pressed at the moment in terms of our appointments…’ (Doctor, Hospital B).

Within this framework, they felt that they still were able to maintain a sufficient flexibility and clinical freedom, as the following example of using the standard form of the discharge letter illustrated:

‘I feel, that one simply can use this [the standard form] as a framework. One uses those parts that are relevant and one ignores the rest. This is at least what I do.’ (Doctor, Hospital B)

The nurses went even further and emphasised that the discharge arrangements could help prevent professional mistakes.

‘It happens from time to time that patients come home and do not really know the plan [for further treatment/control] and that this [plan] has not been put together. Then there occur professional mistakes, and I feel this [discharge arrangements] is really good at preventing this kind of thing [professional mistakes] from happening.’ (Nurse, Hospital B)

The nurse connected the discharge arrangements to the core of their professional practice, namely to avoid mistakes.

In addition, both doctors and nurses suggested that discharge arrangements indirectly were consistent with their professional interests and they referred to the advantages for both patients and general practitioners. This was specifically related to communication, which was seen as a means to safeguard better continuity. The secretaries adopted a similar stance, but, in contrast to doctors and nurses, for the secretaries this was the only connection between discharge arrangements and their professional interests.

In sum, all professional groups felt that discharge arrangements corresponded well with their professional interests and here doctors and nurses, especially, saw a direct connection. In contrast to Hospital A, doctors as well as nurses and secretaries were prepared to mobilise some power resources to pursue their professional interests when they experienced uncertainties about the work flow. At the beginning of the project the doctors and nurses were uncertain about whose responsibility it was to identify possible patients for discharge, and, like in Hospital A, the secretaries did not know how to deal with incomplete standard forms for discharge letters.

In relation to the first issue, management initially defined this as the sole responsibility of doctors, but subsequently redefined this as a responsibility of nurses as well. This mobilised the interests of nurses as the following quote illustrated:

‘Then this [identifying potential patients for discharge] became [an area of] collaboration. This is absolutely fine, […] but this [identifying patients for discharge] also has to be based on collaboration, […] and that it is not only me [who identifies patients]; because otherwise this [the collaboration] becomes meaningless.’ (Nurse, Hospital B)

Importantly, the nurses did not reject the additional task as such, but rather the notion that the task became exclusively their responsibility. This led to the mobilisation of some collaborative power resources where nurses expressed their views in different ways and whereby their interests gradually emerged in an iterative process. The nurses used a log book provided by the senior nurse to note the reluctance of some doctors to share responsibility for identifying potential patients. Importantly, the issues noted in the log book were discussed at the meetings of the project team, which included the leading doctor. As part of this process of collaborative powering, doctors got nurses to take on the task of identifying patients, while accepting the role of nurses as process coordinators.

The secretaries used the joint staff morning meetings with the nurses to mobilise collaborative power resources. The secretaries raised their concerns on the understanding that the leading nurse would pass on any concerns to the project team. The secretaries also used the meetings to clarify their interests, for example when they involved the nurses in a discussion about whether they should simply cut and paste the text dictated by the doctors to fit the standard form. Interestingly, it was agreed that this would go beyond the professional competencies of the secretaries.

#### Process of organisational change: actively involving professional groups

From the analysis, the professional groups emerged as having positive, direct professional interests in discharge arrangement and as willing to mobilise some collaborative power resources when doubts about division of labour emerged. This offered a spring board for professional groups to play a comparatively active role in the process of introducing and routinising discharge arrangements. This occurred in various ways, especially through feeding into formal support by management (which thereby became highly demand led), but also through informal processes of interpreting and adapting the local model as part of regular staff meetings, notably as part of using a log book and as part of individual professional practice.

The initial introduction to the project occurred as part of formal meetings, but, like Hospital A, the individual professional groups were uncertain about the details of the meetings. In contrast to Hospital A, the subsequent support by management, especially the leading nurse, seemed to be extensive but strongly demand led. Here, the nurses were particularly active and the secretaries less so, whereas the doctors made little explicit mention of support by management. As one nurse explained:

‘We [the nurses] also had questions. We had a small black book, where we wrote down our questions, because we were also uncertain about this [the project]. […] But [we also noted] what did not go well in relation to the project.’ (Nurse, Hospital B)

Importantly, the log book not only included questions relating to specific aspects of the discharge arrangements, but also ad hoc evaluations of the process of organisational change. The log book was a means for informing management about relevant areas of support and served as an arena for informal processes of interpreting and adapting the local model of discharge arrangements, while the staff meetings transformed these informal processes into formal decisions about changes in the local discharge arrangements.

This analysis suggested that the approach to organisational change in Hospital B was decentralised and actively involved professional groups that delivered input into demand-led support by management and who, through a log book and meetings, engaged in informal processes of interpretation (and to some extend also adaptation). As the project ended, the process of establishing discharge arrangements as a routine seemed to be well underway. The doctors suggested that discharge arrangements had become internalised in the department and saw the transition to the post-project period as unproblematic. At the same time, the fact that day-to-day practice was highly pressurised also seemed to work as a powerful reminder of the importance of retaining discharge arrangements. The impression of an emerging routine was confirmed by another nurse:

‘I feel that very soon, I will stop thinking about it [discharge arrangements] as a project. I will see it [discharge arrangements] as a natural part of my day-to-day practice.’ (Nurse, Hospital B)

Since the organisational changes associated with discharge arrangements were already so much part of the practice of the professional groups, the official end of the project marked less of a break with ongoing practice and was much less important as a milestone in the process of organisational change than in Hospital A. With the emerging routine, typical activities to support the transition to the post-project period became less relevant for holding on to the organisational change. For example, the nurses discussed the revision of the folders with patient information as ‘nice to have’ rather than ‘must have’.

Yet, both the doctors and the nurses acknowledged that the present level of routinisation was vulnerable to staff turn-over especially among doctors. Among the nurses, this led to a discussion about different possibilities for introducing discharge arrangements to new doctors, but, significantly, there was neither a definite conclusion nor an agreement about what concrete steps to be taken. This was echoed by one of the doctors, who said he assumed that the nurses remembered the discharge arrangements and reminded the new doctors about the routine. Indeed, this was confirmed by the nurses. They observed that, as part of a merger with another hospital’s outpatient department, the nurses often acted as ‘process coordinators’ and introduced the new doctors to discharge arrangements. Taken together, this confirmed both the extent to which the local model of discharge arrangements was routinised and the key role of nurses as ‘process coordinators’ in facilitating routinisation in the future.

## Discussion

One strength of our study is that we were able to compare two departments with many contextual similarities: they were part of the same regional healthcare system and related funding arrangements, they had similar patient populations and they also had the same mix of doctors, nurses and secretaries. At the same time, including additional hospitals in other regions, would have captured a greater variety of types of hospitals and, therefore, increased the robustness of the study. Another strength is that the study was embedded in an implementation project, which allowed us to follow the process of organisational change from its early to its later stages. We analysed the emergence of organisational routines two months after the end of the project period, but such processes can take time; studying the sustainment of organisational change ideally requires a time lapse of at least one year. A follow-up study would offer further insights into the relative level of routinisation. Finally, the present study focused on hospital discharge and it would have been interesting to assess if professional groups play a similar role in comparable organisational changes such as clinical pathways and fast track diagnoses, which affect but do not directly target professional practice. This would increase the generalisability of the present study.

Our theoretical point of departure was to look at the hospital departments as professional organisations, and this is also the basis for our final discussion. Other approaches, for example with a focus on differences in systems and organisational contexts or with a focus on overall processes of organisational change would provide additional insights.

Our analysis revealed two very different local models of discharge arrangements for patients with prostate cancer. Hospital A operated with an ‘add-on’ model, which relied on extra resources, special activities and enforced change. This was reflected in the project nurse who played a prominent role in the discharge arrangements, although she was not part of the day-to-day practice of the outpatient department. On the part of the professional groups, the local model involved either taking on new tasks that were added onto existing practice (like nurses sitting in on consultations) or discontinuing existing practice (like secretaries not contacting doctors in case of queries). In contrast, the local model in Hospital B built on existing ways of working, current resources, and perspectives of professional groups, while the local model evolved gradually and became ‘embedded’ in the existing professional practice.

The emergence of two distinct local models revealed differences in the roles of professional groups in terms of their stakes and involvement in the process of organisational change, both in conjunction with support by management. In Hospital A, the professional stakes were low and the professional groups had highly mixed professional interests and mobilised hardly any resources. Not surprisingly, the professional groups engaged very little in informal processes of interpreting and adapting the local model. This was exacerbated by formal processes that did not involve professional groups, but instead focused on control and problem solving. Taken together, the professional groups were detached from the process of organisational change. The picture looked very different in Hospital B, where the professional stakes were high; all professional groups had direct and positive professional interests and this provided a lever for mobilising some resources in the case of uncertainties. Further, in Hospital B management actively invited staff to share their individual experiences. Such workplace reflection has been shown to be important in changing clinical behavior [33] and, together with other forms of leadership support, is pivotal in facilitating change processes [34]. Professional interests together with active management stitmulated organisational learning and offered a catalyst for multiple informal processes of interpreting and adopting the local model.

What are the broader implications of the analysis for understanding the specific roles of professional groups in organisational changes associated with moving to more explicit discharge arrangements? The analysis makes two key points: first, professional interests are an important driver for health professionals to engage in adapting discharge arrangements; and second, professional practice offers a powerful lever for turning new discharge arrangements into organisational routines. Moreover, both can be greatly strengthened by management, actively governing and guiding such developmental processes.

Concerning the first point, the analysis stressed that professional interests are key for giving meaning to individual health professionals. This is significant because meaning is a central characteristic of those health professionals willing to adapt organisational change [35]. The analysis suggests that while professional interests in discharge arrangements could be either indirect, related to the organisation and to patient, or more direct, the latter emerged as most important. Although all professional groups in both hospitals agreed that the discharge arrangements were meaningful for the department and for patients, they strongly disagreed about the relevance for their professional practice. There was a marked difference between the two hospitals and this seemed to have repercussions for the extent to which professional groups engaged in the process of organisational change. Having positive, direct professional interests emerged as an important driver for actively interpreting and adapting the local model as part of both informal and formal processes, and, thereby, turning discharge arrangements into a routine.

At the same time, the substance of professional interests among professional groups varied. Jespersen et al. [25] suggest that the professional interests of doctors are centred around specialisation and individualisation, whereas for nurses a collective orientation and the patient as a whole person are vital. In contrast, the orientation of secretaries is likely to be more task focused. This was underlined by the present study. Doctors in Hospital B for example, defined their professional interests as improving their own practice, whereas the nurses referred to avoiding mistakes when treating patients across sectors. The secretaries, for their part, mentioned mistakes specifically relating to writing discharge letters. Not surprisingly, among the professional groups, the nurses were most actively engaged in the formal processes and acting collectively in informal processes of interpretation and adaptation of the local model. Indeed, nurses emerged as key agents of organisational change.

In contrast, the engagement of the doctors was more individual in nature and directed at specific patient cases, while the engagement of secretaries was focused specific points of uncertainties in the process of writing discharge letters. However, it is important to remember that the construction of meaning is a complex process and is not necessarily fixed [35].

In relation to the second point, the analysis suggests that adaptation was most successful and discharge arrangements are best sustained when embedded in professional practice. Organisational routines are repetitive patterns of multiple actions that typically involve a range of actors [36]. Routines coordinate and simplify complex situations, and thus represent the behavioural infrastructure of any organisation. This makes organisational routines both the condition for and the object of organisational change.

The analysis demonstrates that health services routines are often specifically related to professional practice, rather than more generically to the organisation. As Kirkpatrick and Ackroyd stress [37], health services belong to a specific type of organisation, namely ‘professional organisations’, where organisational structures are produced and reproduced by members of the profession. There are existing practices and people with whom the organisational change like discharge arrangements needs to be compatible [38]. The embedded local model in Hospital B took account of this and the discharge arrangements were integrated into existing professional practice. Connecting new organisational routines to professional practice provides a springboard for routinized organisational change, precisely because it offers ample opportunity for what Jansen et al. call brokering [39]. Through negotiating organisational change, professional groups not only tailor it to local contexts, but also changed their practice. The situation was very different in Hospital A, where the discharge arrangements were simply added onto existing professional practice. This approach offered few incentives for connecting organisational change to professional practice and for establishing the new discharge arrangements as organisational routines.

Timely discharge is a key component of contemporary hospital governance and this raises questions about how to organise the move to more explicit discharge arrangements. The study points to the specific roles that professional groups played in the making of local models and how their active participation affected the introduction and routinsation of discharge arrangements. Future moves to more explicit hospital discharge arrangements, and the introduction of similar organisational changes like clinical pathways or fast track diagnoses that affect but do not directly target professional practice, therefore, need to have a clearer focus on the concerned professional groups. This requires two-fold approach: involving the professional groups in the introduction and routinisation of new work arrangements and building any new arrangements on existing professional practices. The approach is based on an acknowledgement that the organisation of work practices needs to reflect the specific local contexts of individual hospitals rather than generic best practices. This is supported by recent literature on organisational change in health services [33, 27, 40], which stresses the importance of local contexts and that organisational change is highly contingent on the specific organisation and its environment. A careful analysis needs to be carried out to identify professional groups that will have key roles in a change process. If professional interest is absent, any change will be difficult and, therefore, either the organisational change needs to approached in a different way or abandoned altogether. Also, the existing needs of professional practice have to be revealed and used as a starting point for a gradually evolving new professional practice.

## Conclusions

This paper has treated hospitals as professional organisations, where any organisational change is closely tied to the practices of different groups of health care professionals. The study contributes to the literature on the organisation of discharge arrangements and similar organisational arrangements by specifying the crucial role played by professional groups in making such changes. Firstly, professional interests are central for giving meaning to individual health professionals and, as such, emerge as a highly influential driver for health professionals to engage in adapting discharge arrangements to the specific context of the local hospital. Secondly, professional practice also offers a powerful lever for the successful adaption and for turning the new discharge arrangements into organisational routines. Management can stimulate both processes by active support.

## Abbreviations

GPs: General practitioners
PSA: Prostate specific antigen
